# Optimization Design and Performance Analysis of a Bionic Knee Joint Based on the Geared Five-Bar Mechanism

**DOI:** 10.3390/bioengineering10050582

**Published:** 2023-05-11

**Authors:** Zhuo Wang, Wenjie Ge, Yonghong Zhang, Bo Liu, Bin Liu, Shikai Jin, Yuzhu Li

**Affiliations:** School of Mechanical Engineering, Northwestern Polytechnical University, Xi’an 710072, China

**Keywords:** kangaroo knee joint, bionic mechanism, dynamics, energy efficiency

## Abstract

Animal joint motion is a combination of rotation and translational motion, which brings high stability, high energy utilization, and other advantages. At present, the hinge joint is widely used in the legged robot. The simple motion characteristic of the hinge joint rotating around the fixed axis limits the improvement of the robot’s motion performance. In this paper, by imitating the knee joint of a kangaroo, we propose a new bionic geared five-bar knee joint mechanism to improve the energy utilization rate of the legged robot and reduce the required driving power. Firstly, based on image processing technology, the trajectory curve of the instantaneous center of rotation (ICR) of the kangaroo knee joint was quickly obtained. Then, the bionic knee joint was designed by the single-degree-of-freedom geared five-bar mechanism and the parameters for each part of the mechanism were optimized. Finally, based on the inverted pendulum model and the Newton–Euler recursive method, the dynamics model of the single leg of the robot in the landing stage was established, and the influence of the designed bionic knee joint and hinge joint on the robot’s motion performance was compared and analyzed. The proposed bionic geared five-bar knee joint mechanism can more closely track the given trajectory of the total center of mass motion, has abundant motion characteristics, and can effectively reduce the power demand and energy consumption of the robot knee actuators under the high-speed running and jumping gait.

## 1. Introduction

Legged robots, such as bipedal and quadrupedal robots, exoskeletons, and intelligent active prostheses, may be used in field exploration [1,2,3], disaster relief [4,5,6,7], home services [8,9,10], and medical rehabilitation [11,12,13,14], and have the potential to change human life in the future. Many researchers have already conducted a lot of research in the field of legged robots, showing astoundingly excellent results, such as Boston Dynamics’ bipedal robot Atlas, which dances like a real person [15], and the quadrupedal robot ANYmal from ETH Zurich, which takes only 31 min to climb a 120 m high mountain (a total distance of 2.2 km), which is 4 min faster than a human on foot [16]. However, the energy efficiency of the various legged robots that are now publicly available is still significantly lower than that of animals with similar locomotion [17,18], and the input power required to complete their locomotion is extremely high, which leads to the problem caused by the large size and weight of the drive system.

Legged robots usually consist of a control module, a drive module, and a motion executive mechanism composed of links. Some researchers have studied intelligent control methods [19,20,21] and high-performance actuators [22,23,24,25] to improve the motion stability and power density of legged robots with excellent results, but they are now gradually encountering development bottlenecks. Other researchers have started from the optimization design of the executive mechanism [17,26,27,28] to solve the above problems.

The hardware structure of legged robots consists mainly of links that are usually connected by revolute joints, but may also be connected by prismatic joints. With the continuous development in the field of biomechanics, numerous studies on the leg joints of mammals, especially the knee and ankle joints [29,30,31,32], have shown that these two joints consist of irregularly shaped bones with the characteristic of multi-axis rotation, that is, their instantaneous center of rotation (ICR) trajectory presents a J-shaped curve. Such joints have greater foot clearance and smaller flexion angles than the uniaxial joints commonly used in legged robots, as well as better ground reaction forces to maintain motion stability. Revolute joints and prismatic joints with simple movement rules have difficulty in imitating the movement characteristics of biological joints, so they cannot obtain the high efficiency and high stability of biological locomotion.

To improve the bionic properties of the joints in legged robots, researchers have proposed various innovative mechanisms to achieve the motion patterns of the biological knee and ankle joints. In the field of robotics, A. Hamon designed the knee joint of a biped robot with the four-bar mechanism [33] and the crossed four-bar mechanism [34], which reduced the energy consumption of the robot and the impact of the foot against the ground during walking. Alexander G mimicked the design of the human knee joint by introducing a single sliding link and replicating the condylar surfaces of the femur and tibia [35]. Yixiang L used planetary gear transmission [36] to achieve changes in the instantaneous center of rotation of the designed knee joint, which outperformed the traditional hinge knee joint in terms of the range of motion and power consumption. However, the design basis of these joint mechanisms, i.e., the ICR curves of the knee and ankle joints of the referenced organisms, were obtained by measuring the parameters of the bone shapes at the biological joints. The period of measurement and processing of experimental data is very long, so it is difficult to realize the fast and automatic design process in the bionic joint design for different bone shapes (such as different bionic objects). At present, the technology for acquiring the surface contour of the target object by image processing technology has a certain foundation, and the application of image processing technology to acquire the surface contour of the target joint is expected to achieve the rapid determination of the optimization target for the design of the bionic joint mechanism and, thus, speed up the design process.

In the field of mechanical exoskeletons and prostheses, Michał O designed a new adjustable knee joint mechanism based on a cross four-bar mechanism [37] by introducing two additional degrees of freedom, which can achieve a variety of knee trajectories. Yonghong Z applied gait acquisition technology to design a six-bar mechanism prosthesis knee joint [38], which can help amputees to accomplish a variety of gait patterns. The main design principle of these joint mechanisms is to make the motion curves of the designed joints fit the actual human knee and ankle motion patterns as much as possible, which facilitates the designed exoskeleton and prosthesis to precisely follow the human lower limb motion. Legged locomotion is mainly divided into walking, running, jumping, and other modes. Humans only have high stability and energy utilization in low-speed walking mode, while in high-speed running, jumping, and other movement modes, cheetahs, kangaroos, and other creatures have obvious advantages, their joints are excellent bionic objects, which may be able to improve the high-speed mobility and energy utilization efficiency of legged robots during running and jumping.

Moreover, current studies mainly discuss the influence of different knee joints on the kinematic performance of bipedal or quadrupedal robots in walking locomotion mode, such as knee extension, foot clearance, and energy consumption [35,36,39,40]. Research on the kinematic performance of mechanical joints based on ICR trajectories of biological joints during high-speed gaits (running and jumping) has not been published, which limits the further application of bionic joints in legged robots.

In the real wild world, kangaroos have the advantages of fast locomotion, robust jumping motion, and low energy consumption compared to other running and jumping quadrupeds. When a kangaroo reaches a certain jumping speed, its oxygen consumption is lower than that of a quadruped with the same mass and speed [41]. During high-speed gaits, such as running and jumping, it has been shown that the knee joint provides the greatest torque and plays a major role in energy consumption [42]. In this paper, by imitating and researching the motion of the kangaroo knee joint, the knee joint mechanism of the legged robot is designed based on the planar multi-bar mechanism, to improve the joint trajectory richness of the legged robot, while reducing the energy cost of the robot actuators during the high-speed gait. The following are the main contributions from the paper:

- Rapid acquisition for the ICR trajectory of the knee joint. Based on the image of the kangaroo leg bone, the contact curve between the kangaroo femur and tibia was automatically acquired using a high-order polynomial fitting method with computer image processing technology;
- Kangaroo-inspired knee joint mechanism design and optimization. The bionic knee joint was designed by a geared five-bar (GFB) mechanism with a single-degree-of-freedom and the parameters for each part of the mechanism were optimized. The ICR trajectory of the kangaroo knee joint was accurately tracked, and the rotation angles of the thigh and tibia in the designed mechanism were consistent with the motion data on the kangaroo lower limb;
- Performance analysis of the GFB joint during high-speed gaits. Based on the spring inverted pendulum model, the Newton–Euler recursive method was used to establish the dynamics model of the single leg of the robot in the landing stage and the centroid trajectory of the robot model was planned. The effects of the hinge joint mechanism and the GFB joint mechanism on the required driving power and energy consumption of the robot at high speed were compared and analyzed.

The remainder of this paper is organized as follows: In Section 2, we acquire the ICR trajectory of the kangaroo knee joint, and design and optimize the GFB mechanism to accurately track this trajectory; in Section 3, a single-leg model of the robot with the GFB knee joint and hinge knee joint is constructed, and the kinematic performance of these two mechanisms is compared and analyzed; in Section 4, the required driving power and energy of the two knee joints are analyzed; finally, the conclusions and other reflections from the paper are provided in Section 5.

## 2. The Design and Modeling of a New Bionic Knee Joint Mechanism

### 2.1. The ICR Analysis of the Knee Joint of the Australian Grey Kangaroo

In this section, the knee joint of an Australian grey kangaroo is studied. Kangaroos are typically jumping animals that can travel long distances at speeds of up to 50 km/h. During a long journey, kangaroo hopping has a low energy consumption [41]. This advantage may be related to the motion characteristics of the kangaroo leg joints. We studied the knee bones of the grey kangaroo to obtain the ICR curve of the femur relative to the tibia. Apparently, the skin, muscles, and bones in the kangaroo’s legs move periodically during jumping, and their movement characteristics are different. Compared with skin and muscles, bones are more similar to rigid bodies, and pictures of leg bones are easy to obtain. Therefore, studying the skeleton picture is a convenient way to obtain the ICR curve.

**Figure 1:**
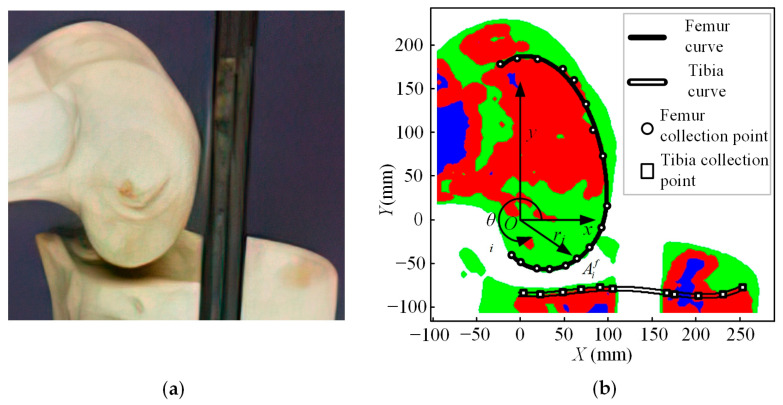
(**a**) Grey kangaroo knee joint bone photo; (**b**) grey kangaroo knee joint bone RGB image.

**Figure 2:**
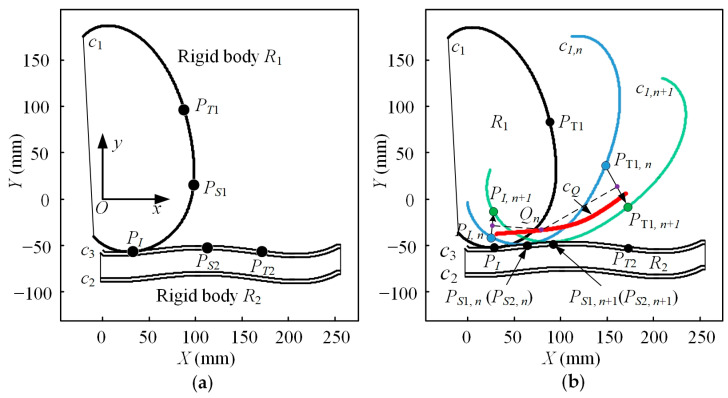
(**a**) The schematic diagram of an equivalent rigid body of knee joint bones; (**b**) ICR curve analysis.

The skeleton picture (Figure 1a) of the kangaroo knee joint used in this paper is from an adult eastern Australian grey kangaroo (Macropus giganteus), which belongs to the skeletal specimen collection in the Australian Museum [43]. In Figure 1a, the condyles of the femur and tibia of the kangaroo are complete, grayish white, with a clear surface contour, and accurate relative position. In Figure 1a, there is a metal bracket blocking the tibia and the photo must be processed to obtain a clearer image. To make the data obtained after image processing more accurate, we enlarge the original image four times, that is, the scale of Figure 1 and Figure 2 is 4:1. We use the image processing toolbox in MATLAB to process the original photos into RGB images of the knee joint bones (Figure 1b), and use the *ginput* function to conveniently collect the contact curves of the femur and tibia.

In Figure 1b, the tibial curve can be obtained using a polynomial fitting method for the tibial acquisition points in the Cartesian coordinate system. The tibial curve equation is as follows:

(1) $$y_{t}=f_{t}\left(x_{t}\right)=\sum_{j=0}^{4}P_{j}^{t}x_{t}{}^{j},$$

where $P_{j}^{t}$ is the polynomial coefficient, *x_t_* and *y_t_* are the x-coordinate value and y-coordinate value of the point on the fitted curve, and $x_{t}\in \left[x_{min},x_{max}\right]$. Moreover, *x_min_* is the minimum x-coordinate value of the tibial collection point, and *x_max_* is the maximum x-coordinate value of the tibial collection point. The degree of the sum polynomial is 4.

The femoral collection point in Figure 1b cannot fit a polynomial in a Cartesian coordinate system, but it can fit a polynomial in a polar coordinate system. The fitting polynomial of the femoral curve is detailed below.

This is example 1 of an equation:

(2) $$r_{f}=f_{f}\left(\theta_{f}\right)=\sum_{k=0}^{6}P_{k}^{f}\theta_{f}{}^{k},$$

where $P_{k}^{f}$ is the polynomial coefficient, *θ_f_* is the polar angle of a point on the curve, and *r_f_* is the corresponding polar diameter. The degree of the sum polynomial is 6.

In Figure 1b, the curve of the femur and the curve of the tibia are not in contact. The reason is that in addition to the condyle of the femur and the condyle of the tibia, the knee joint of the kangaroo also contains patella, meniscus, and synovium, etc., which have been removed from the bone specimens. Among them, patella and synovium have almost no influence on the contact surface of the kangaroo knee joint, so they are not considered in the subsequent calculation. The condyle of the femur is covered with a layer of hyaline articular cartilage. The shape of the articular cartilage is consistent with the shape of the articular surface of the bone and the articular cartilage covering the femoral condyle is very thin. Therefore, the external contour curve of the femoral condyle is used as the contact curve of the upper part of the kangaroo knee joint in this paper.

The condyle of the tibia is covered with a cushioning fibrous cartilage called the meniscus. The meniscus thickness is usually more than 3 mm and, therefore, cannot be ignored in subsequent ICR curve calculations. According to the anatomical study of the kangaroo knee joint conducted by the University of Western Australia [44], the contact area between the femoral condyle and meniscus is mainly located in the central area of the meniscus, where the contour curve of the upper surface of the meniscus is basically parallel to that of the tibial condyle. Therefore, this paper introduces a virtual meniscus curve as the lower contact curve of the kangaroo knee joint. This curve can be obtained by translating the tibial curve along the positive *y*-axis (Figure 2). The polynomial of the meniscus curve is:

(3) $$y_{m}=f_{m}\left(x_{m}\right)=f_{t}\left(x_{t}\right)+b=\sum_{j=0}^{4}P_{j}^{m}x_{m}{}^{j},$$

where $P_{j}^{m}$ is the polynomial coefficient, *x_m_* and *y_m_* are the coordinates of the point on the curve *c*_3_, and *b* is the displacement of the curve *c*_3_ relative to the curve *c*_2_ in the positive direction of the *y*-axis.

Both the femur curve and tibia curve can be regarded as rigid bodies, and the law of relative motion of the two curves can be regarded as the law of relative motion of the femur and tibia. Before calculating the ICR curve, the movement law of the femur curve relative to the tibia curve must be strictly defined.

In Figure 2a, the rigid body *R*_1_ is the femur, and the rigid body *R*_2_ is the tibia. The definition of the movement law is as follows: there are moving points *P_S_*_1_, *P_S_*_2_, and fixed points *P_I_*, *P_T_*_1_, and *P_T_*_2_ on the curves *c*_1_ and *c*_3_, respectively. The two moving points can move along their respective curves. The fixed point *P_I_* is the starting point of the movement of *P_S_*_1_ and *P_S_*_2_, and the fixed points *P_T_*_1_ and *P_T_*_2_ are the endpoints of the movement of *P_S_*_1_ and *P_S_*_2_, respectively. Moreover, *θ_T1_* the polar angle value of the fixed point *P_T_*_1_ is greater than *θ_I_* the polar angle value of the fixed point *P_I_*, and $x_{T1}$ the x-coordinate value of *P_T_*_2_ is greater than $x_{I}$ the x-coordinate value of *P_I_*. The displacement of points *P_S_*_1_ and *P_S_*_2_ at time *t* can be expressed as $S_{2}=f_{S,2}(t)$, where $t\in \left[0,t_{E}\right]$, and *t_E_* is the end time of the movement. At any time *t* during the full-period motion, the rigid body *R*_2_ is fixed to the fixed reference system, and *P_S_*_1_ on the rigid body *R*_1_ coincides with *P_S_*_2_ on *R*_2_. There is no overlap between *R*_1_ and *R*_2_, and curve *c*_1_ on *R*_1_ is tangent to curve *c*_3_ on *R*_2_.

In Figure 2b, *c*_1_ is the femoral curve, *c*_2_ is the tibial curve, and c_3_ is the meniscus curve. The blue curve and green curve with the same shape as curve c1 but in different positions are denoted as *c*_1,n_ and *c*_1,n+1_, respectively. They are different states of the femur curve during the movement of the kangaroo knee joint. Similar to Figure 2a, *P*_S1,n_ and *P*_S2,n_ are the moving points on curve *c*_1,n_ and curve *c*_3_, respectively, and *P*_I,n_ and *P*_T1,n_ represent the starting point and ending point of the moving point *P*_S1,n_ on curve *c*_1,n_. The definition for each point on the curve *c*_1,n+1_ is similar to that of the curves *c*_1_ and *c*_1,n_, except that the subscript of each point is n + 1. The red curve is the calculated ICR curve and *Q*_n_ is the discrete point on the curve.

According to the above description, the displacement functions *f_S_*_1_ and *f_S_*_2_ of the points *P_S_*_1_ and *P_S_*_2_ can be used to determine the unique pose of the rigid body *R*_1_ relative to the absolute reference frame at time *t*, that is to say, the rolling and sliding of the rigid body *R*_1_ relative to the rigid body *R*_2_ can be converted into the movement of points *P_S_*_1_ and *P_S_*_2_ along the curves *c*_1_ and *c*_3_.

If the motion laws of points *P_S_*_1_ and *P_S_*_2_ on curves *c*_1_ and *c*_3_ are the same, according to the above definition, the pose of the rigid body *R*_1_ at any time can be obtained. The discrete ICR point *Q_n_* of the femur relative to the tibia can be calculated according to the vertical bisectors of the points *P_I_*_,*n*_, *P_I_*_,*n*+1_, *P_T_*_1,*n*_, and *P_T_*_1,*n*+1_ in two adjacent frames, where *n* is the number of frames. The ICR curve *c_Q_* is the connection of all the discrete ICR points.

### 2.2. The Design and Optimization of the Bionic GFB Knee Joint Mechanism

**Figure 3:**
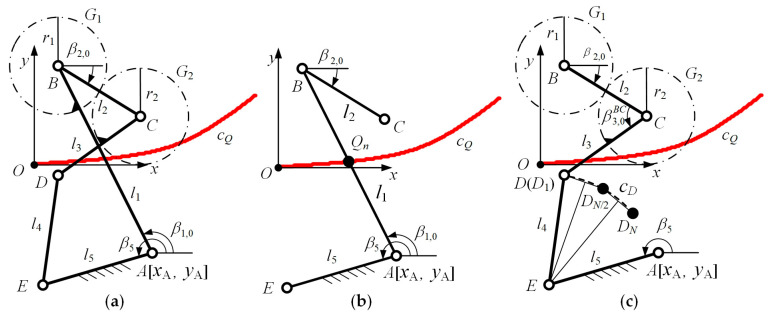
The optimization process for the GFB mechanism: (**a**) definition of the coordinate system and key parameters of the GFB mechanism; (**b**) the open-chain three-bar mechanism to determine the configuration parameters; (**c**) the open-chain four-bar mechanism to determine the remaining parameters.

As shown in Figure 3a, the plane GFB mechanism consists of five connecting bars *AB*, *BC*, *CD*, *DE*, and *AE*, and two gears *G*_1_ and *G*_2_. The bar *AE* is fixedly connected to the tibial rigid body *R*_2_ and fixedly connected to the coordinate system *Oxy*. The bar *BC* is fixedly connected to the femur rigid body *R*_1_. The origin of the coordinate system *Oxy* is at point *Q*_1_ of the ICR curve *c_Q_*. Moreover, *x_A_* and *y_A_* are the absolute coordinate values of point *A*. Angle $\beta_{1,0}$ is the initial angle of bar *AB* and the angle $\beta_{2,0}$ is the initial angle of bar *BC*. In addition,$\beta_{5}$ is the angle of the fixed bar *AE*. Furthermore, *r*_1_ is the index circle radius of gear *G*_1_, *r*_2_ is the index circle radius of gear *G*_2_, and the transmission ratio of the two gears is $\lambda =r_{1}/r_{2}$.

For the GFB mechanism to simulate the kinematic characteristics of the kangaroo knee joint, not only must the ICR point of the bar *BC* relative to the bar *AE* be on the curve *c_Q_* at any time, but also the rotation angle of the bar *BC* must be the same as the kangaroo’s femur. Obviously, this is a multi-variable and multi-objective optimization problem. Using the Kennedy–Aronhold theorem can effectively reduce the number of variables and the objectives in the optimization problem of GFB mechanisms. We split the GFB mechanism shown in Figure 3a into a planar open-chain three-bar mechanism and a planar open-chain four-bar mechanism, thus transforming the optimization problem into two single-objective optimization problems for analysis.

As shown in Figure 3b, the open chain three-bar mechanism is composed of bars *AE*, *AB*, and *BC*. At any number of frames *n*, the bar *AB* or its extension line must pass through the point *Q_n_*, and the rotation angle of the bar *BC* is not related to [*l*_2_, *l*_5_, *β*_2,0_, *β*_5_]. The process for determining the configuration parameters [*x*_A_, *y*_A_, *l*_1_] of the mechanism is a three variable single-objective optimization problem. According to the Kennedy–Aronhold theorem, the optimization objective function is:

(4) $$F=\min\left(f\left(x_{A},y_{A},l_{1}\right)\right)=\min\left(\frac{\sum_{n=1}^{N}{\left(\Delta \phi_{BC,n}\left(x_{A},y_{A},l_{1},n\right)-\left(\phi_{R1,n}-\phi_{R1,n-1}\right)\right)}^{2}}{N}\right),$$

where $∆\emptyset_{BC,n}$ is the angle the bar BC has turned, and it is also a function of [*x*_A_, *y*_A_, *l*_1_, n]. Moreover,$\emptyset_{R1,n}$ and $\emptyset_{R1,n-1}$ are the angles the rigid body *R*_1_ has turned at frame *n* and *n*−1. *N* is the total amount of frames. To avoid the bar length being too large or too small, the boundary condition of the bar length *l*_1_ is specified as:

(5) $$30\;mm<l_{1}<300\;mm,$$

According to function (4) and boundary condition (5), the *fmincon* function is used to find the minimum value of *f*(*x*_A_, *y*_A_, *l*_1_). The algorithm is the interior-point algorithm, and the tolerance of the variable iteration and the function value are all 10^−6^. Given a suitable initial value, the objective function converges to the point [40.54, −70.08, 244], where [40.54, −70.08] is the coordinate of point *A*, and 244 is the bar length *l*_1_ of bar *AB*. The function value at the convergence point is 7.85 × 10^−7^.

According to the value of the parameter [*x*_A_, *y*_A_, *l*_1_], the rotation angle changes $∆\emptyset_{AB,n}$ and $∆\emptyset_{BC,n}$ of bar *AB* and bar *BC* can be calculated. Then, the open-chain four-bar mechanism shown in Figure 3c should be analyzed. When the number of frames is *n*, the rotation angles of bar *BC* and bar *CD* relative to the absolute coordinate system are:

(6) $$\begin{array}{l} \phi_{BC,n}=\beta_{2,0}+\sum_{i=1}^{n}\Delta \phi_{BC,n}\\ \phi_{CD,n}=\phi_{BC,n}+\pi +\beta_{3,0}^{BC}+\frac{\sum_{i=1}^{n}\Delta \phi_{BC,n}-\Delta \phi_{AB,n}}{\lambda } \end{array},$$

where $\beta_{3,0}^{BC}$ is the initial angle of bar *CD* relative to bar *BC*. Further, the coordinates of point *C* and point *D* are:

(7) $$\begin{array}{l} x_{C,n}=x_{A}+l_{1}\cos\left(\sum_{i=1}^{n}\Delta \phi_{AB,n}\right)+l_{2}\cos\phi_{BC,n}\\ y_{C,n}=y_{A}+l_{1}\sin\left(\sum_{i=1}^{n}\Delta \phi_{AB,n}\right)+l_{2}\sin\phi_{BC,n}\\ x_{D,n}=x_{C,n}+l_{3}\cos\phi_{CD,n}\\ y_{D,n}=y_{C,n}+l_{3}\sin\phi_{CD,n} \end{array},$$

When the GFB mechanism moves from the frame number *n* = 1 to *n* = *N*, the discrete positions of the hinge point D will form a curve *c_D_*. Point *E* is a fixed point relative to the absolute coordinate system, and the bar length *l*_4_ of bar *DE* is constant. Therefore, if the curve *c_D_* is a circular arc with point *E* as the center, the GFB mechanism can simulate the ICR curve and the rotation angle law of the kangaroo knee joint. The objective function of the second optimization of the mechanical structure is:

(8) $$\begin{array}{ll} F & =\min\left(f\left(l_{2},l_{3},\beta_{2,0},\beta_{3,0}^{BC}\right)\right)\\ & =\min\left(\begin{array}{l} \frac{\sum_{n=1}^{N}{\left(\sqrt{{\left(x_{D,n}-x_{E}\right)}^{2}+{\left(y_{D,n}-y_{E}\right)}^{2}}-\frac{\sum_{n=1}^{N}\sqrt{{\left(x_{D,n}-x_{E}\right)}^{2}+{\left(y_{D,n}-y_{E}\right)}^{2}}}{N}\right)}^{2}}{N} \end{array}\right) \end{array},$$

To ensure that the angle values are all within the range of [0, 2π), the boundary conditions of *β*_2,0_ and $\beta_{3,0}^{BC}$ are:

(9) $${\left[\beta_{2,0},\beta_{3,0}^{BC}\right]}^{T}\in \left[{\left[0,0\right]}^{T},{\left[2\pi ,2\pi \right]}^{T}\right),$$

In addition, the length of each bar should not be too large or too small, so the boundary conditions of the bar length are given as follows:

(10) $${\left[l_{2},l_{3}\right]}^{T}\in \left[{\left[30,30\right]}^{T},{\left[300,300\right]}^{T}\right],\;\left[\begin{array}{l} \sqrt{{\left(x_{D,n}-x_{E}\right)}^{2}+{\left(y_{D,n}-y_{E}\right)}^{2}}\\ \sqrt{{\left(x_{A}-x_{E}\right)}^{2}+{\left(y_{A}-y_{E}\right)}^{2}} \end{array}\right]\in \left[\begin{array}{c} \left[30,300\right]\\ \left[30,300\right] \end{array}\right],$$

We have calculated the convergence value of the objective function when *λ* takes different values, and the optimization effect is best if *λ* = 2. When each parameter takes the value shown in Table 1, the objective function (3) converges, and the function value at the convergence point is 1.36 × 10^−7^. 

**Figure 4:**
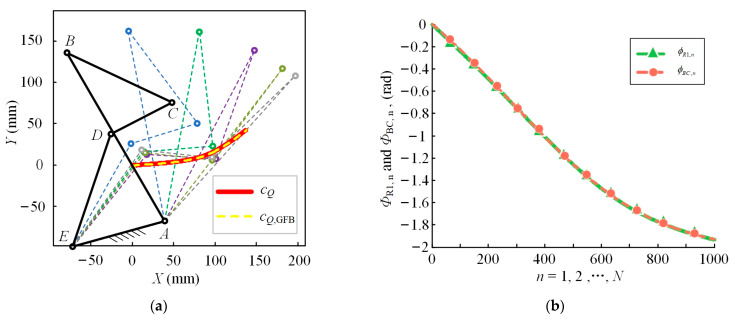
(**a**) The ICR curves of the GFB mechanism and the kangaroo; (**b**) the rotation angle trajectory of the GFB mechanism and the kangaroo.

The solid black line in Figure 4a shows the initial configuration of the GFB mechanism and the dashed lines in other colors show the different states. The red solid line is the actual ICR curve of the kangaroo knee joint referenced in the paper and the yellow dashed line is the trajectory of the ICR curve achieved by the designed GFB mechanism. As shown in the figure, these two curves have the same change trend and basically coincide. In Figure 4b, we fix the tibia of the kangaroo, and the rotation angles of the femur during motion is *Φ*_R1,n_, i.e., the green solid line. The whole moving process is divided into 1000 equal parts, corresponding to the horizontal coordinates in Figure 4b. In the GFB mechanism, bar AE corresponds to the tibia of the kangaroo and bar BC corresponds to the femur of the kangaroo. The orange dashed line represents the rotation angle of the bar BC. The maximum angle difference between these two curves occurs at the position corresponding to the value 101 of the transverse coordinate, which is 0.0062 rad. The GFB mechanism fits the ICR curve and rotation angle law of the kangaroo knee joint well, which proves the accuracy of the kangaroo knee joint ICR curve acquisition technology and the bionic knee joint mechanism design method proposed in this paper.

## 3. The Analysis and Discussion of the Inverse Kinematics

It is difficult to explain the influence of the proposed GFB mechanism on the motion performance and driving demand of the legged robot by analyzing the GFB knee joint alone. Therefore, it is necessary to establish a general robot model for comparative analysis. At present, hinge joints are the mainstream design scheme for legged robot knee joints, such as Boston Dynamics’ bipedal robot Atlas [15] and IIT’s bipedal robot WALK-MAN [45], and ETH’s quadrupedal robot ANYmal [1], etc., all of which adopt hinge knee joints. The bipedal robot Cassia [46] and the quadrupedal robot Cheetah [47] from the MIT, transfer their driving motors to the hip using a parallelogram mechanism at the knee joint, which is essentially a hinged knee joint. To explain the influence of the GFB mechanism on the motion performance of the legged robot, we constructed two simplified models of the robot leg with a GFB knee joint and a hinge knee joint. Then, the kinematic performance of these two mechanisms is compared and analyzed. We found that when the size and quality parameters of the main components are similar, there is almost no difference in the total center of gravity (TCM) working space of the two robot mechanisms through forward kinematics analysis. Therefore, the following sections focus on the analysis of the inverse kinematics characteristics of the two mechanisms.

### 3.1. The Modeling of the Single Leg for Robots

**Figure 5:**
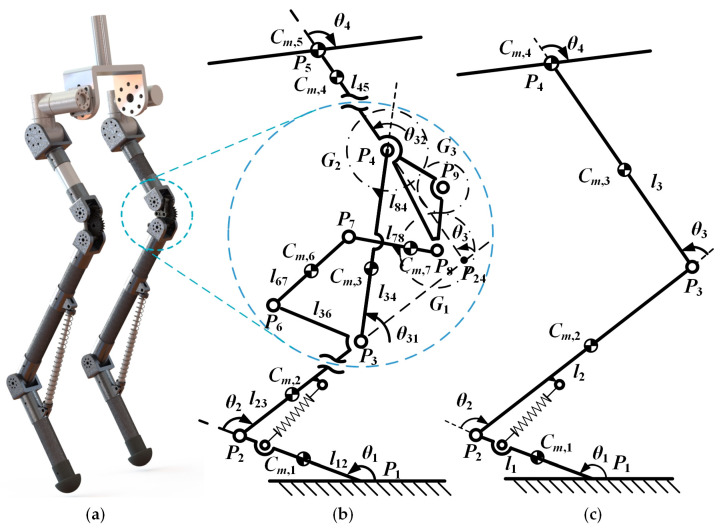
The schematic diagrams of the robotic mechanical structure with the GFB knee joint and the hinge knee joint: (**a**) 3D model of the legged robot; (**b**) the single-leg model of the robot with a GFB knee joint; (**c**) the single-leg model of the robot with a hinge knee joint.

According to the optimization results of the GFB mechanism in Section 2, the bars *AB* and *CD* have a positive transmission ratio (Figure 3). There should be three gears articulated with bar *BC*. The schematic model of the robot mechanisms can be established based on the parameter values of each component, given in Table 1, and the measurement data of the length of the kangaroo femur and tibia and the size parameters of the knee joint bone. The three-dimensional model of the designed legged robot is shown in Figure 5a, which mainly contains the body, thigh, knee joint, shank, foot, and other parts. The hip joint at the connection between the thigh and the body, as well as the knee joint at the connection between the thigh and the shank, are actively driven by motors, while the ankle joint at the connection between the shank and foot is passively driven by a spring.

Figure 5b is a single-leg model of the robot with a GFB knee joint mechanism. For clarity, the mechanism of the knee joint is partially enlarged. Point *P*_1_ is the contact point between the toe and the ground. Points *P*_2_ to *P*_8_ are the hinges between the components. *G*_1_, *G*_2_, and *G*_3_ are the gears. Moreover, *G*_1_ and *G*_2_ are fixedly connected with bar *P*_7_*P*_8_ and bar *P*_3_*P*_4_, respectively. Gear *G*_3_ and bar *P*_4_*P*_8_ are hinged at point *P*_9_. Points *C_m_*_,1_ to *C_m_*_,7_ are the center points of mass (CM) of the components, and CM *C_m_*_,1_ and hinge *P*_5_ are coincident. Bar *P*_2_*P*_3_ and bar *P*_3_*P*_6_ are fixedly connected, and bar *P*_4_*P*_5_ and bar *P*_4_*P*_8_ are fixedly connected. The length between the hinge points on each member is represented by *l*, for example, *l*_12_ is the length from points *P*_1_ to *P*_2_. Point *P*_1_ is the intersection of bar *P*_2_*P*_3_ and the *P*_4_*P*_5_ extension. Angle *θ*_1_ to *θ*_4_ is the rotation angle variables of the joint space, and the knee joint angle *θ*_3_ is the angle from bar *P*_2_*P*_3_ to bar *P*_4_*P*_5_. In addition, *θ*_31_ is the angle from bar *P*_2_*P*_3_ to bar *P*_3_*P*_4_, and *θ*_32_ is the angle from bar *P*_3_*P*_4_ to bar *P*_4_*P*_5_. Figure 5c is a single-leg model of the robot with a hinge knee joint mechanism, and the meaning of each symbol is similar to Figure 5b.

In Figure 5b, since the masses of bars *P*_3_*P*_4_, *P*_6_*P*_7_, and *P*_7_*P*_8_ at the knee joint account for a small proportion of the total mass of the robot, it can be considered that there is only one bar *P*_3_*P*_4_ with mass properties in the knee joint, and the mass of bar *P*_3_*P*_4_ is the sum of the actual masses of bars *P*_3_*P*_4_, *P*_6_*P*_7_, and *P*_7_*P*_8_. In addition, to facilitate the calculation, a polynomial fitting method is used to establish the relationship between angles *θ*_3_, *θ*_31_, and *θ*_32_, and the polynomial is as follows:

(11) $$\theta_{31}=\sum_{i=1}^{4}A_{i}\theta_{3}{}^{i-1},\;\theta_{32}=\theta_{3}-\theta_{31},$$

where ***A*** = [−31.35, 0.3927, 4.306 × 10^−3^, −1.968 × 10^−5^]. The root mean square (RMSE) of the polynomial is 0.0797 and the coefficient of determination (R-square) is 1.

According to the DH method, the position of the TCM of the model with a GFB mechanism is:

(12) xm0=(m1+m2+m3+m4+m5)−1[m1m2m3m4m5][x1cm,1c1−y1cm,1s1l12c1+x2cm,2c1,2−y2cm,2s1,2l12c1+l23c1,2+x3cm,3c1,2,31−y3cm,3s1,2,31l12c1+l23c1,2+l34c1,2,31+x4cm,4c1,2,31,32−y4cm,4s1,2,31,32l12c1+l23c1,2+l34c1,2,31+l45c1,2,31,32+x5cm,5c1,2,31,32,4−y5cm,5s1,2,31,32,4],

(13) y0m=(m1+m2+m3+m4+m5)−1[m1m2m3m4m5][x1cm,1s1+y1cm,1c1l12s1+x2cm,2s1,2+y2cm,2c1,2l12s1+l23s1,2+x3cm,3s1,2,31+y3cm,3c1,2,31l12s1+l23s1,2+l34s1,2,31+x4cm,4s1,2,31,32+y4cm,4c1,2,31,32l12s1+l23s1,2+l34s1,2,31+l45s1,2,31,32+x5cm,5s1,2,31,32,4+y5cm,5c1,2,31,32,4],

where *s*_1,2,31_ and *c*_1,2,31_ are the sine and cosine of the sum of angles *θ*_1_, *θ*_2_, and *θ*_31_, respectively. Other similar symbols indicate similar meanings. The TCM position of the model with a hinge joint mechanism is similar to (13).

### 3.2. The Analysis of the TCM Trajectory

**Figure 6:**
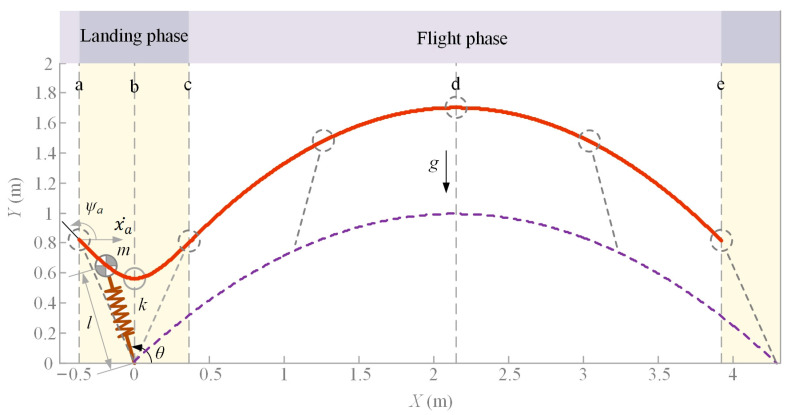
The SLIP model.

A large number of studies show that the running and jumping motion can be described by a spring-loaded inverted pendulum (SLIP) model. To carry out the inverse kinematics, it is necessary to plan the trajectory of the centroid and the endpoint of the foot using the SLIP model. Since the knee joint angle of the legged robot in the flight phase is almost unchanged, and the active joints consume little energy in the flight phase, we only analyze the landing phase (the yellow area in Figure 6) of the SLIP model.

As shown in Figure 6, the CM trajectory (the red line) of the symmetric SLIP model is taken as the robot TCM motion trajectory. Where *m* is the mass of the CM, *g* is the acceleration of gravity, *l* is the length from the CM to the toe, and *k* is the spring stiffness. The direction of the CM horizontal velocity ${\dot{x}}_{a}$ is the positive direction of the *x*-axis. The model enters the landing phase at position *a*. The CM reaches its lowest point at position *b*. The spring is fully extended at position *c*, and the model takes off. Moreover, *ψ_a_* is the angle between the CM velocity and the horizontal direction at the beginning of the landing phase, and *θ* is the angle between the spring axis and the horizontal direction. 

During the landing phase, the Euler–Lagrange equation for the CM is:

(14) $$L=T-V=\frac{1}{2}m\left({\dot{l}}^{2}+l^{2}{\dot{\theta }}^{2}\right)-mgl\sin\theta -\frac{1}{2}k{\left(l_{a}-l\right)}^{2},$$

The equation of motion for the CM is:

(15) $$\{\begin{array}{l} ml^{2}\ddot{\theta }+2ml\dot{l}\dot{\theta }-mgl\cos\theta =0\\ m\ddot{l}-ml{\dot{\theta }}^{2}+mg\sin\theta -k\left(l_{a}-l\right)=0 \end{array},$$

The state vector of the CM is:

(16) $$\{\begin{array}{l} p={\left[\begin{array}{cccc} \theta & \dot{\theta } & l & \dot{l} \end{array}\right]}^{T}\\ \dot{p}=\left[\begin{array}{l} \dot{\theta }\\ \frac{g\cos\theta }{l}-\frac{2\dot{l}\dot{\theta }}{l}\\ \dot{l}\\ l{\dot{\theta }}^{2}-g\sin\theta +\frac{k}{m}\left(l_{a}-l\right) \end{array}\right] \end{array},$$

The given parameters and initial values are as follows, *m* = 20 kg, *g* = 9.8 m/s^2^, the free length of the spring is *l_a_* = 0.850 m, the initial horizontal velocity is ${\dot{x}}_{a}$ = 4.167 m/s, *ψ_a_* = 135°, and the initial angle between the spring axis and the horizontal direction is *θ_a_* = 111.8°. The differential Equations (15) are accurately solved with the *ode45* function in MATLAB, and the trajectory of the CM is shown in Figure 6.

### 3.3. Inverse Kinematics Analysis

To facilitate the inverse kinematics analysis of the robot model, (13) is simplified, and the robot TCM position calculation formula is as follows:

(17) x0m=a1c1+a2c1,2+a3c1,2,31+a4c1,2,3y0m=a1s1+a2s1,2+a3s1,2,31+a4s1,2,3a1=l12(M−0.5m1)M,a2=l23(M−m1−0.5m2)Ma3=l34(0.5m3+m4+m5)M,a4=m4l45/2+m5l45M,

where *M* is the total mass of the robotic model. Additionally, (17) is further simplified by the trigonometric function formula and the simplified form is:

(18) x0m=a1c1+(a2+a3c31+a4c3)c1,2−(a3s31+a4s3)s1,2y0m=a1s1+(a2+a3c31+a4c3)s1,2+(a3s31+a4s3)c1,2A=x0m(a2+a3c31+a4c3)+y0m(a3s31+a4s3)B=x0m(a3s31+a4s3)−y0m(a2+a3c31+a4c3)C=x0m2+y0m2+(a2+a3c31+a4c3)2+(a3s31+a4s3)2−a12D=a2+a3c31+a4c3E=a3s31+a4s3,

when the knee angle *θ*_3_ and the CM coordinates [^0^*x*_m_, ^0^*y*_m_] are given. Moreover, *θ*_31_ can be obtained with (11), and *A*, *B*, *C*, *D,* and *E* in (18) can be calculated. The sine and cosine values of *θ*_1_, *θ*_1,2_, *θ*_31_, and *θ*_3_ are:

(19) [c3s3c31s31c1,2s1,2c1s1]=[c3s3c31s31AC±|B|4A2−C2+4B22(A2+B2)2Ac1,2−C2B[x0m−Dc1,2+Es1,2]/a1[y0m−Ds1,2−Ec1,2]/a1],

The angle values for *θ*_1_, *θ*_1,2_, *θ*_31_, and *θ*_3_ can be obtained with the four-quadrant arctangent function atan2. Finally, *θ*_1_, *θ*_2_, *θ*_31_, and *θ*_32_ can be obtained according to the relationship between the joint angle, and selected according to:

(20) $$\{\begin{array}{l} {\left[\theta_{1}\;\theta_{2}\right]}^{T}\geq {\left[45^{\circ }\;-120^{\circ }\right]}^{T}\\ {\left[\theta_{1}\;\theta_{2}\right]}^{T}\leq {\left[172^{\circ }\;-0.5^{\circ }\right]}^{T} \end{array},$$

The solution process for the robot model with the hinge joint, shown in Figure 5c, to track the CM motion trajectory of the SLIP model is similar to the above process.

**Figure 7:**
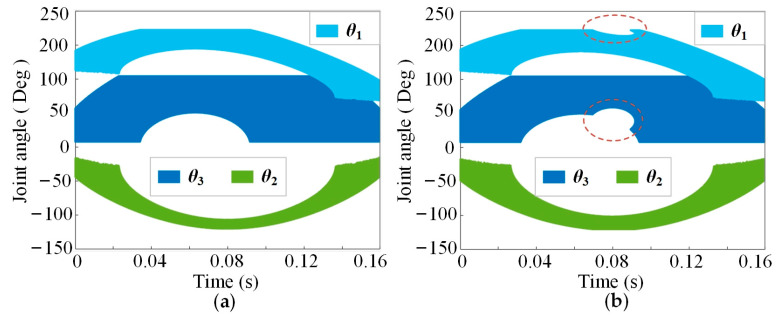
The solution spaces of the joint angles: (**a**) the robot with the GFB joint; (**b**) the robot with the hinge joint.

The solution spaces of the joint angles in the two robot models that track the known TCM trajectories are shown in Figure 7a,b. Obviously, in the region selected by the dotted line of the red ellipse in Figure 7b, the feasible region of the inverse kinematics solution shrinks, which does not appear in Figure 7a. Compared with the hinge knee robot, the GFB knee robot contains more optional angles for each joint when tracking the TCM trajectory shown in Figure 6, which makes it easier to avoid possible collision with the surrounding environment during the landing phase. In other words, the GFB knee robot can run and jump more flexibly. This is an advantage of the GFB knee joint in robot inverse kinematics.

## 4. The Analysis and Discussion of the Inverse Dynamics

### 4.1. The Modeling of the Inverse Dynamics

At present, Lagrangian equations are generally used to analyze the inverse dynamics of robots. Compared with the Lagrangian equation, the dynamic analysis using the Newton–Euler recursive method is not only more efficient but can also directly obtain the force of each component in the joint space.

**Figure 8:**
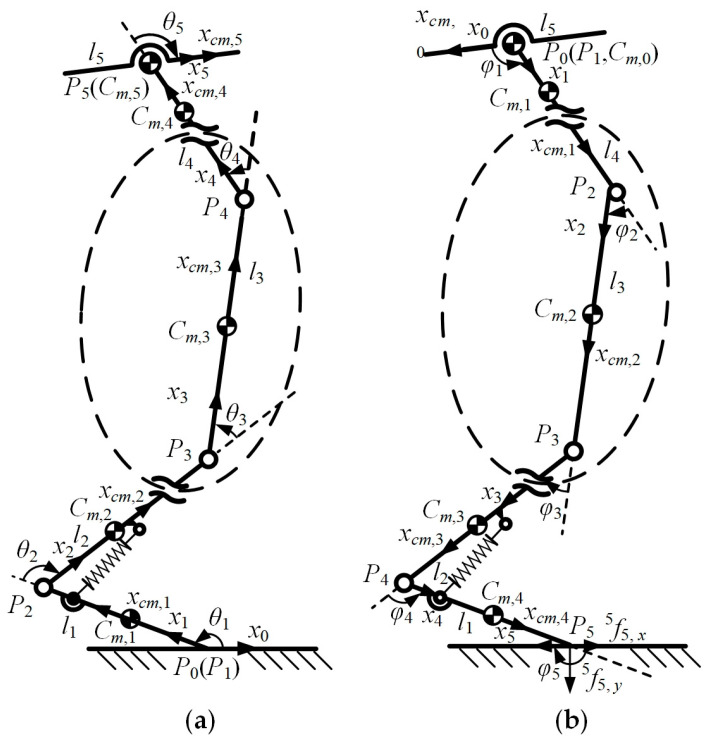
The coordinate systems and variables definition for the robotic models: (**a**) the extrapolation process; (**b**) the interpolation process.

As shown in Figure 8a, since the masses of bars *P*_6_*P*_7_ and *P*_7_*P*_8_ are added to bar *P*_3_*P*_4_, the part of the knee joint framed by the elliptical dotted line is simplified to only one bar *P*_3_*P*_4_. Moreover, *x*_0_, *x*_1_, … *x*_5_ are the x-coordinate axes of the joint space coordinate system of the robot model, and *x*_0_ is the *x*-axis of the base coordinate system. In addition, *x_cm_*_,1_, *x _cm_*_,2_, … *x _cm_*_,5_ are the x-coordinate axes of the CM space coordinate system. The direction of the *z*-axis of each coordinate system is outward and the direction of the *y*-axis is determined according to the right-hand rule. The meanings of the other symbols are the same as in Figure 5b.

The symbol definitions are given as follows: {*i*} represents the joint space coordinate system, and *i* is the serial number of each bar, where *i* = 0, 1, …, 4. In addition, ^i^***ν***_i_ and ^i^***ω***_i_ are the linear and angular velocity of the joint space coordinate system, respectively. Furthermore, *^i^**P**_i_*_+1_ is the coordinate vector of the origin of the coordinate system {*i* + 1} in the coordinate system {*i*}. Then, *θ_i_*_+1_ is the angle between bar *i* + 1 and bar *i*.

The angular velocity of the bar *i* + 1 in the coordinate system {*i*} is:

(21) ωii+1=ωii+Ri+1iθ˙i+1Z^i+1i+1,

where Ri+1i is the rotation matrix from the coordinate system {*i*} to {*i* + 1}. The above formula is transformed into the following form with the left multiplied by Ri+1i:

(22) ωi+1i+1=Rii+1ωii+θ˙i+1Z^i+1i+1,

The linear velocity of bar *i* + 1 in the coordinate system {*i*} is:

(23) vii+1=vii+ωii×Pii+1,

The transformation is:

(24) vi+1i+1=Rii+1(vii+ωii×Pii+1),

Considering that the *z*-axis of each coordinate system in the two-dimensional plane is always outward, (14) can be transformed into:

(25) ωi+1i+1=ωii+θ˙i+1Z^i+1i+1,

The first derivative with respect to time *t* is:

(26) ω˙i+1i+1=ω˙ii+θ¨i+1Z^i+1i+1,

The tangential linear acceleration of the coordinate system {i + 1} in {i} is:

(27) v˙ii+1t=ω˙ii×Pii+1,

Then, the normal acceleration is:

(28) v˙ii+1n=ωii×(ωii×Pii+1),

The linear acceleration of bar *i* + 1 in the coordinate system {*i*} is:

(29) v˙ii+1=ω˙ii×Pii+1+ωii×(ωii×Pii+1)+v˙ii,

The transformation with the left multiplied by Rii+1 is:

(30) v˙i+1i+1=Rii+1[ω˙ii×Pii+1+ωii×(ωii×Pii+1)+v˙ii],

The CM linear acceleration of bar *i* + 1 in the coordinate system {*i* + 1} is:

(31) v˙i+1cm,i+1=ω˙i+1i+1×Pi+1cm,i+1+ωi+1i+1×(ωi+1i+1×Pi+1cm,i+1)+v˙i+1i+1,

Given the linear acceleration and angular acceleration at the CM of bar *i* + 1, the Newton–Euler dynamic equation for bar i + 1 is:

(32) Fi+1i+1=mi+1v˙i+1cm,i+1Ni+1i+1=Icm,i+1i+1ω˙i+1i+1+ωi+1i+1×Icm,i+1i+1ωi+1i+1=Icm,i+1i+1ω˙i+1i+1,

where ^i+1^***F***_i+1_, ^i+1^***N***_i+1_, and ^cm,j+1^***I***_i+1_ are the force, the torque, and the moment of inertia of bar *i* + 1 at the CM of itself, respectively, and the value of ^i+1^***ω***_i+1_ × ^cm,i+1^*I*_i+1_ ^i+1^***ω***_i+1_ in the two-dimensional plane is 0. The formulas (25), (26), …, (32) constitute the extrapolation calculation process for the Newton–Euler recursive method.

### 4.2. Interpolation Process of the Dynamics

For an open-chain robot, a single bar has the following dynamic balance of force and torque in the CM space:

(33) Fii=fii−Ri+1ifi+1i+1Nii=nii−nii+1+(−Picm,i)×fii−(Pii+1−Picm,i)×fii+1,

where ^i^***n***_i_ and ^i^***n***_i+1_ are the joint torque of bar *i* in the coordinate system {*i* + 1} and {*i*}, respectively, ^i^***f***_i_ and ^i^***f***_i+1_ are the joint force of bar *i* in the coordinate system {*i* + 1} and {*i*}, respectively, and ^i^***P***_cm,i_ is the coordinate vector of the CM in the coordinate system {*i* + 1}. If ^i^***f***_i+1_ and ^i^***n***_i+1_ are left multiplied by the rotation matrix Ri+1i, the transformation of ^i^***N***_i_ is:

(34) Nii=nii−Ri+1ini+1i+1−Picm,i×fii−Pii+1×Ri+1ifi+1i+1+Picm,i×Ri+1ifi+1i+1=nii−Ri+1ini+1i+1−Picm,i×Fii−Pii+1×Ri+1ifi+1i+1,

The force and torque dynamic balance of the bar in the joint space are:

(35) fii=Ri+1ifi+1i+1+Fiinii=Nii+Ri+1ini+1i+1+Picm,i×Fii+Pii+1×Ri+1ifi+1i+1,

The torque of bar *I* at joint *i* is:

(36) τi=niiTZ^ii,

Different from the interpolation process of the robotic arm, there is only force between the toe of the legged robot and the ground, but no torque. Therefore, the interpolation process of the robotic arm dynamics cannot be directly applied. It is worth noting that the recurrence formula for the extrapolation process does not include the force and the motion at the joints, and the interpolation process does not include the angular velocity, the angular acceleration, and the linear acceleration. Without considering the rotation matrices Ri+1i and Rii+1, the Newton–Euler extrapolation process and the interpolation process are independent of each other. Therefore, considering that the structure, the motion, and the force characteristics of the robotics are different, the coordinate system definitions for the extrapolation method and the interpolation method can be different when performing recursive operations.

Fixing the base coordinate system at the joint of the femur bar, the fixed coordinate system of each bar is redefined as shown in Figure 8b, and *φ*_1_, *φ*_2_, … *φ*_5_ are the redefined joint angles. Each CM coordinate system is rotated 180° around the *z*-axis. Moreover,Ri+1i, Rii+1, ^i^***F***_i_, and ^i^***N***_i_ are written in the new form in the redefined coordinate system, and *i* = 4, 3, …, 1. Then, (35) and (36) can be used to calculate the joint space force and torque of each bar.

### 4.3. The Required Drive Torque of the Knee Joint

Section 4.1 and Section 4.2 in this paper have given the calculation methods for the force and torque of each main bar of the GFB joint robot, but the GFB knee joint also contains two bars *P*_6_*P*_7_ and *P*_7_*P*_8_ that do not count the mass.

**Figure 9:**
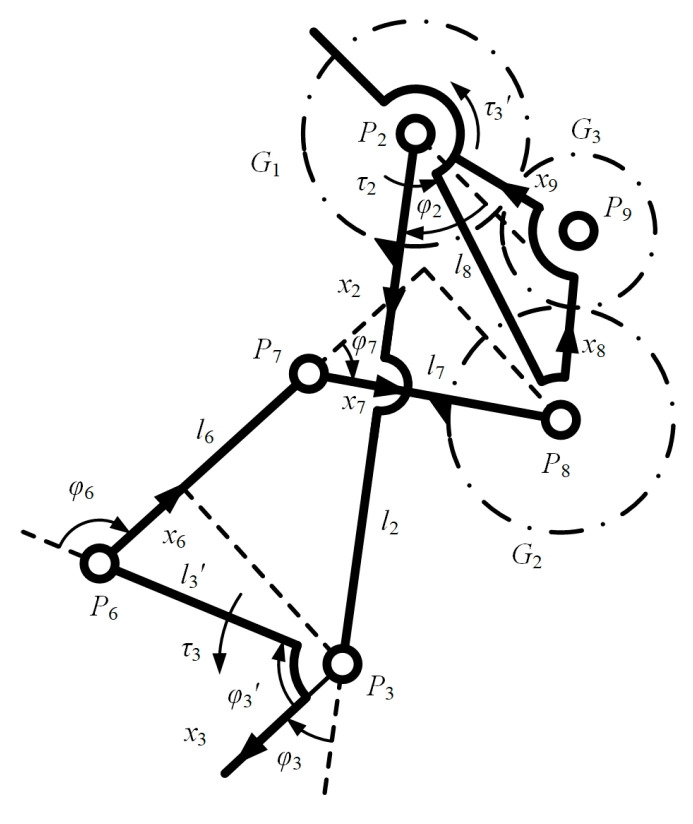
The coordinate systems and variables definition for the GFB mechanism.

As shown in Figure 9, *φ*_3_*’*, *φ*_6_, and *φ*_7_ are the joint angles, and *l*_3_*’*, *l*_6_, and *l*_7_ are the length of each bar. 

The torque at joint *P*_3_ of the bar *P*_2_*P*_3_ is not generated by a physical actuator, but instead is supplied by the force transmitted from the real actuator at joint *P*_4_ to bars *P*_6_*P*_7_ and *P*_7_*P*_8_. According to the Newton–Euler static equation, the additional torque that the actuator at joint *P*_4_ needs to provide is:

(37) $$\tau_{3}{}^{\prime }=\frac{\tau_{3}l_{7}s_{7}^{\varphi }}{\lambda_{23}\lambda_{31}l_{3}{}^{\prime }s_{6}^{\varphi }},$$

where *λ*_23_ and *λ*_31_ are all the gear ratios. The total torque provided at joint *P*_4_ is:

(38) $$\tau_{2}^{Total}=\tau_{2}+\tau_{3}{}^{\prime }=\tau_{2}+\frac{2\tau_{3}l_{7}s_{7}^{\varphi }}{l_{3}{}^{\prime }s_{6}^{\varphi }},$$

### 4.4. The Analysis of the Energy Cost and Power Requirement

In Figure 7, two sets of joint angle change trajectories belonging to the GFB joint robot and the hinge joint robot are selected as the inverse dynamic calculation variables. The knee joint angle trajectories for these two groups of angle trajectories are the same. By inputting variables into (25), (26), …, (38), we can obtain the inverse dynamics of the GFB joint robot and the hinge joint robot. If the drive torque at the initial moment of the robotic landing phase does work as ***W***_i,0_ = 0, the values of work for each joint actuator are:

(39) $$\tau_{2}^{Total}=\tau_{2}+\tau_{3}{}^{\prime }=\tau_{2}+\frac{2\tau_{3}l_{7}s_{7}^{\varphi }}{l_{3}{}^{\prime }s_{6}^{\varphi }},$$

The driving power of each joint is:

(40) $$\tau_{2}^{Total}=\tau_{2}+\tau_{3}{}^{\prime }=\tau_{2}+\frac{2\tau_{3}l_{7}s_{7}^{\varphi }}{l_{3}{}^{\prime }s_{6}^{\varphi }},$$

**Figure 10:**
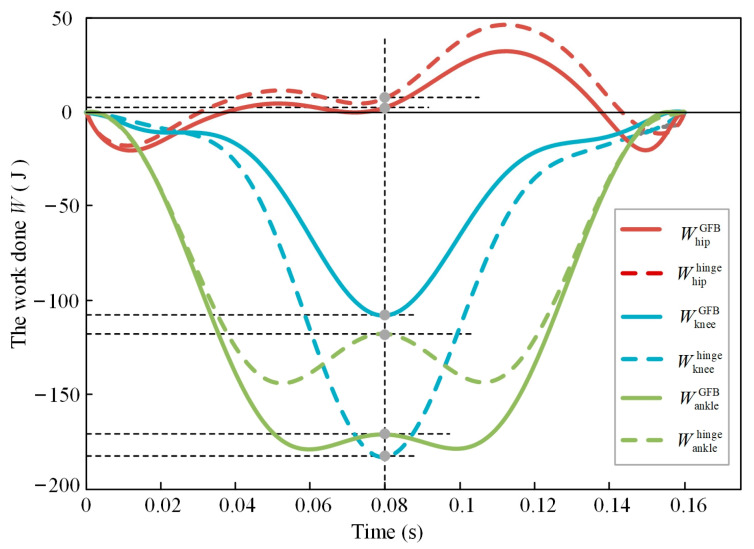
The energy consumption of the joint actuators.

As shown in Figure 6 and Figure 10, the TCMs of the two robots are located at the lowest point at 0.08 s, when the landing phase is symmetrically divided into two parts. At this moment, the total kinetic energy and potential energy of the robot reach the valley value, the elastic potential energy stored in the spring reaches the peak value, and the work conducted by each joint is representative. For the GFB joint model, the hip joint drive work is $W_{hip}^{GFB}$ = 2.7 J, the knee joint drive work is $W_{knee}^{GFB}$ = −108.5 J, and the ankle joint drive work is $W_{ankle}^{GFB}$ = −172.6 J. For the hinge joint model, the hip joint drive work is $W_{hip}^{hinge}$ = 7.7 J, the knee joint drive work is $W_{knee}^{hinge}$ = −184.3 J, and the ankle joint drive work is $W_{ankle}^{hinge}$ = −118.6 J. The hip and knee joints are driven by active actuators, and the ankle joints are driven by springs. The energy input requirement for the active actuator at the lowest TCM point can be obtained by adding the work of the hip and knee joints. The energy input requirement for the GFB model to the active actuator is 59.91% of the hinge joint model. It can be considered that the GFB knee joint mechanism can transfer the energy cost from the active actuator to the ankle joint elastic passive actuator, and the maximum transmission ratio is about 40.09%. 

**Figure 11:**
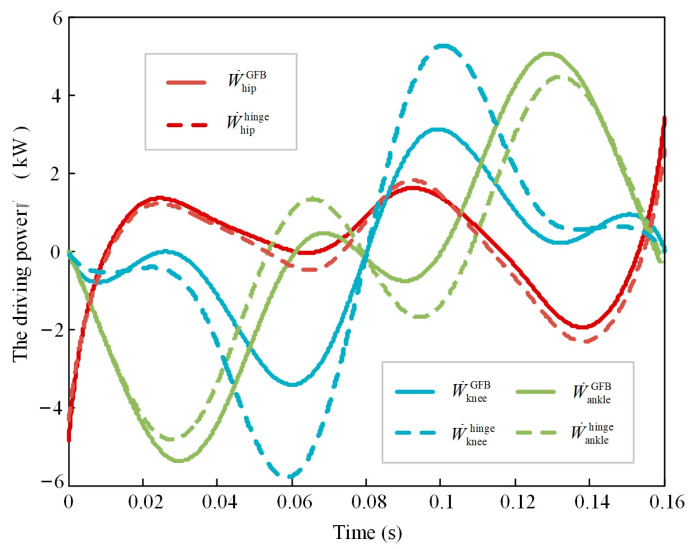
The required power of the joint actuators.

Figure 11 shows the power demand curves for the two robot models during the landing phase. The maximum absolute values for the active actuator power cost of the GFB joint model and the hinge joint model are 5.104 kW and 5.766 kW, respectively, and the ratio is 88.52%. The ranges of active actuator power cost for the two models are 9.446 kW and 10.83 kW, and the ratio of the GFB model to the hinge model is 87.22%. Furthermore, the standard deviation for the active actuator power cost is:

(41) $$\sigma =\sqrt{\frac{\sum_{j=1}^{n}{\left({\dot{W}}_{Total}^{j}-\frac{\sum_{j=1}^{n}{\dot{W}}_{Total}^{j}}{n}\right)}^{2}}{n}},$$

where *j* is the serial number of the frames of motion, and *n* is the total number of frames. The standard deviation for the active actuator power cost of the GFB model is 2.279 kW, the standard deviation for the active actuator power cost of the hinge model is 3.435 kW, and the ratio is 66.35%. Therefore, the GFB mechanism can effectively reduce the active actuator power cost of the legged robot.

## 5. Conclusions

To improve the bionic performance and motion ability of the legged robot, a kangaroo-inspired knee joint based on the geared five-bar mechanism is proposed. The bionic knee joint designed in this paper can accurately track the ICR trajectory of the kangaroo knee joint, and the rotation angles of the bars representing the thigh and shank in the mechanism are consistent with the movement law of the kangaroo’s lower limb. This verifies the effectiveness of the rapid acquisition of the kangaroo knee bone contour and the optimization design method of the knee joint mechanism in this paper. The TCM trajectory in the landing phase of the robot is planned using the SLIP model, and the influence of the GFB knee joint and the hinge joint on the motion performance of the robot is investigated by applying the Newton–Euler iteration method. Compared with the hinged knee joint, the GFB knee joint has a larger feasible solution space for the joint angle, that is, the legged robot equipped with the GFB knee joint has more alternative postures and configurations during motion. The GFB knee joint described in this paper can effectively reduce the energy consumption and power required in legged robots. The compact GFB joint can also be applied to other legged motion systems with multiple joints, such as prostheses, exoskeletons, industrial manipulators, etc., which are expected to improve their dynamic performance and reduce power consumption.

The single leg of the robot studied in this paper is not only a legged robot, but also an independent motion module in a multi-legged robot. Therefore, the GFB knee joint mechanism with richer motion trajectories and lower energy consumption can also be applied to the leg mechanism of a multi-legged robot. In addition, the mechanism model shown in Figure 5b can be regarded as a manipulator arm with a fixed base, the bar P1P2 is regarded as the base, the joint P2 is regarded as the base joint, the circle dotted area is regarded as the elbow joint, and joint P5 is regarded as a wrist joint. In this way, the GFB elbow joint mechanism can reduce the power requirements of the wrist and elbow joints on the drive and transfer the energy consumption of the wrist and elbow joint drive to the base joint drive.

## Figures and Tables

**Table 1:** The parameters of the GFB mechanism when *λ* = 2.

Variable	Value	Variable	Value
*l*_1_ (mm)	244.00	*x_A_* (mm)	40.54
*l*_2_ (mm)	145.90	*y_A_* (mm)	−70.08
*l*_3_ (mm)	87.20	*Β*_1,0_ (rad)	2.0952
*l*_4_ (mm)	149.50	*β*_2,0_ (rad)	0.4396
*l*_5_ (mm)	120.20	*β*_5_ (rad)	−2.8709

## Data Availability

Not applicable.
